# Membrane androgen receptor activation triggers down-regulation of PI-3K/Akt/NF-kappaB activity and induces apoptotic responses via Bad, FasL and caspase-3 in DU145 prostate cancer cells

**DOI:** 10.1186/1476-4598-7-88

**Published:** 2008-12-03

**Authors:** Natalia Papadopoulou, Ioannis Charalampopoulos, Vasileia Anagnostopoulou, Georgios Konstantinidis, Michael Föller, Achilleas Gravanis, Konstantinos Alevizopoulos, Florian Lang, Christos Stournaras

**Affiliations:** 1 Department of Biochemistry, University of Crete Medical School, Heraklion, Greece; 2 Department of Pharmacology, University of Crete Medical School, Heraklion, Greece; 3 Department of Physiology, University of Tübingen Medical School, Tübingen, Germany; 4 Medexis-Biotech SA, Kryoneri, Athens, Greece

## Abstract

**Background:**

Recently we have reported membrane androgen receptors-induced apoptotic regression of prostate cancer cells regulated by Rho/ROCK/actin signaling. In the present study we explored the specificity of these receptors and we analyzed downstream effectors controlling survival and apoptosis in hormone refractory DU145-prostate cancer cells stimulated with membrane androgen receptor-selective agonists.

**Results:**

Using membrane impermeable conjugates of serum albumin covalently linked to testosterone, we show here down-regulation of the activity of pro-survival gene products, namely PI-3K/Akt and NF-κB, in DU145 cells. Testosterone-albumin conjugates further induced FasL expression. A FasL blocking peptide abrogated membrane androgen receptors-dependent apoptosis. In addition, testosterone-albumin conjugates increased caspase-3 and Bad protein activity. The actin cytoskeleton drug cytochalasin B and the ROCK inhibitor Y-27632 inhibited FasL induction and caspase-3 activation, indicating that the newly identified Rho/Rock/actin signaling may regulate the downstream pro-apoptotic effectors in DU145 cells. Finally, other steroids or steroid-albumin conjugates did not interfere with these receptors indicating testosterone specificity.

**Conclusion:**

Collectively, our results provide novel mechanistic insights pointing to specific pro-apoptotic molecules controlling membrane androgen receptors-induced apoptotic regression of prostate cancer cells and corroborate previously published observations on the potential use of membrane androgen receptor-agonists as novel anti-tumor agents in prostate cancer.

## Background

An increasing body of scientific evidence points to the existence of two types of androgen receptors: (a) intracellular androgen receptors (iARs) mediating genomic androgen signals resulting in receptor dimerization, nuclear translocation and subsequent activation of androgen-specific target genes (reviewed in [1]) and (b) membrane androgen receptors (mARs) triggering non-genomic signals manifested within minutes of androgen binding (reviewed in [2,3]). Although the exact molecular identity of mAR still remains unknown, it is believed that mAR may represent either (I) a pool of iAR targeted to the plasma membrane and/or associated membrane structures (e.g. lipid rafts or caveolae) mediating rapid androgen effects in the absence of transcriptional activity (reviewed in [4]) or (II) an unknown G-protein coupled receptor (GPCR) (or a receptor associated with a GPCR) triggering a variety of iAR-independent signaling cascades. These cascades typically result in increased intracellular [Ca^2+^]_i _and inositol 1,4,5-triphosphate formation, are sensitive to pertussis toxin inhibition [5,6] and cannot be blocked by anti-androgens [7,8]. Rapid, non-genomic androgen actions have been reported in various cell types including macrophages and T cells [9,10], LNCaP [7], T47D [11], MCF7 [12], DU145 [13], C6 [14], PC12 [15] or VSMC cells [16].

We and others have recently characterized mAR-dependent signaling events in prostate and breast cancer cell lines [8,11,12,17]. Using non-permeable androgen derivatives that do not bind to iAR, namely conjugates of testosterone covalently linked to bovine serum albumin (testosterone-BSA), we have specifically shown that activation of mAR results in actin reorganization of iAR^+^/mAR^+ ^LNCaP and iAR^-^/mAR^+ ^DU145 prostate cancer cell lines [8,17]. Furthermore, we have shown that testosterone-BSA induces apoptotic regression of LNCaP and DU145 cells *in vitro *and in mouse xenografts *in vivo *[13,18]. Finally, testosterone-BSA suppresses cell motility and potentiates paclitaxel-mediated cytotoxicity both *in vitro *and *in vivo *[12,18]. However, the specific pro-apoptotic molecules controlling the mAR-induced apoptosis in prostate cancer cells remained unknown.

In the present study we have analyzed the specificity of mAR and the activity of downstream gene products playing a prominent role in survival and apoptosis in DU145 prostate cancer cells. Our results show that testosterone-BSA suppresses PI-3K activity, inhibits Akt function and finally inactivates the pro-survival transcription factor NF-κB. We further report mAR-dependent suppression in the phosphorylation/inactivation of the pro-apoptotic Bad protein, stimulation of FasL expression and induction of caspase-3 activity. Taken together, our results provide new mechanistic insight into specific mAR-dependent apoptosis of prostate cancer cells.

## Materials and methods

### Cell culture and transfections

The DU145 human prostate cancer cell line was obtained from the American Type Culture Collection (Manassas, VA) and was studied between passages 60 and 70. DU145 cells fail to respond to androgen treatment owing to the expression of non-functional iAR [19], or to complete lack of iAR according to other studies [20,21].

### Preparation of steroid solution

Before each experiment testosterone-3-(O-carboxymethyl) oxime-BSA, referred to as testosterone-BSA, dihydrotestosterone (DHT), estradiol-BSA and dexamethasone (Sigma), were dissolved in serum-free culture medium at a final concentration of 10^-5 ^M. The steroid-albumin conjugates-stock solutions were incubated for 30 min at room temperature with 0.3% charcoal and 0.03% dextran, centrifuged at 3000 × g and passed through a 0.45 μm filter to remove any potential contamination with free steroid. Testosterone-BSA, estradiol-BSA, dexamethasone and DHT solutions were used at a final concentration of 10^-7 ^M throughout all studies. If not otherwise stated all treatments and incubations with steroids including apoptosis assays were performed in serum-containing medium.

### Measurement of F/G actin ratio by Triton X-100 fractionation

The Triton X-100 soluble G-actin containing and insoluble F-actin containing fractions of cells exposed to testosterone-BSA and DHT were prepared as previously described [22]. An increase of the triton-insoluble (F) to triton-soluble (G) actin ratio is indicative of actin polymerization.

### Immunoprecipitation and Western blot analysis

DU145 cells treated or not (control cells) with testosterone-BSA were washed twice with ice-cold phosphate-buffered saline and suspended in cold lysis buffer containing 1% Nonidet P-40, 20 mM Tris (pH 7.4) and 137 mM NaCl, supplemented with protease and phosphatase inhibitors. Cleared lysates were pre-adsorbed with protein A-Sepharose beads (Amersham) for 1 h at 4°C. Equal amounts of the supernatants were subjected to immunoprecipitation using an anti-phosphotyrosine (PY20) antibody (Santa Cruz Biotechnology) and protein A-Sepharose beads. For immunoblot analysis the immunoprecipitates and equal amounts of total protein extracts were suspended in Laemmli's sample buffer and separated by SDS-PAGE. For Fas ligand expression studies cells were pretreated or not with 10^-7 ^M cytochalasin B (Biomol Research Laboratories, PA), or 10 μM Y-27632 (Calbiochem), and stimulated with 10^-7 ^M testosterone-BSA for the time periods indicated in the figure legends.

Proteins were transferred onto nitrocellulose membranes and blotted with rabbit polyclonal anti-PI-3K p85 (Upstate) (1:1000 dilution), rabbit polyclonal anti-phospho-Akt Ser473, anti-phospho-Akt Thr308 or anti-Akt (total) (Cell Signaling) (1:500 dilution), rabbit polyclonal anti-Fas-L (Q20, Santa Cruz) (1:200 dilution), phospho- and total Bad antibodies (Santa Cruz Biotechnology, Santa Cruz, CA, USA, 1:1000 dilution). Secondary antibodies used were horseradish peroxidase-conjugated anti-mouse IgG (Chemicon), and horseradish peroxidase-conjugated anti-rabbit IgG (Immunotech, France). Then, the membranes were exposed to Kodak X-Omat AR films. A PC-based Image Analysis program was used to quantify the intensity of each band (Image Analysis, Inc., Ontario, Canada).

### NF-κB Transcription factor Assay

A non-radioactive NF-κB p50/p65 Transcription Factor Assay was used to detect specific transcription factor DNA binding activity in nuclear extracts (Chemicon, San Diego, CA, USA). A double stranded biotinylated oligonucleotide containing the consensus sequence for NF-κB binding (5'-GGGACTTTCC-3'), was mixed with cellular (nuclear) extract pre-treated or not with 10^-7 ^M cytochalasin B. After co-incubation the active form of NF-κB contained in the nuclear extract binds to its consensus sequence. Thereafter, the extract/probe/buffer mixture was directly transferred to the streptavidin-coated plate. The biotinylated double stranded oligonucleotide bound by active NF-κB protein was immobilized, and any inactive unbound material was washed away. The bound NF-κB transcription factor subunits, p50/p65, were detected with specific primary antibodies. An HRP-conjugated secondary antibody was then used for detection and quantification in a spectrophotometric plate reader. By loading the same amount of total protein in each sample, we ensured the exact normalization for all cases.

#### Measurement of apoptosis

##### APOPercentage apoptosis assay

DU145 cells (in RPMI 1640, supplemented with 25 mM HEPES, 2 mM L-Glutamine and 10% FBS) were cultured in 96-well plates for the *APOPercentage apoptosis assay *(Biocolor Ltd., Belfast, Ireland). In the presence or absence of 10^-7 ^M flutamide (Sigma), cells were stimulated or not with 10^-7 ^M of the following steroids in serum-supplemented medium: testosterone-BSA (Testo-BSA), dihydrotestosterone (DHT), estradiol-BSA (E2-BSA) and dexamethasone (DEXA) or 10^-7 ^M BSA for 24 hours. Untreated cells cultured in serum free medium were used as positive control for the apoptotic response.

##### FACS analysis

DU145 cells (in RPMI 1640, supplemented with 25 mM HEPES, 2 mM L-Glutamine and 10% FBS) were cultured in 60 mm plates for FACS analysis and determination of Fas expression levels. After pre-treatment with a monoclonal Ab to Fas, (Fas-blocking peptide, 805-C10-C100, Alexis Biochemicals, Axxora LLC, San Diego, USA), cells were stimulated or not with 10^-7 ^M testosterone-BSA in serum-supplemented medium for the time periods indicated in the figure legends. Untreated cells cultured in serum-free medium were used as a positive control for the apoptotic response. At the end of the respective treatment cells were harvested in PBS and stained with the Annexin V-FITC Apoptosis Detection kit I (BD Pharmingen TM, San Diego, CA) according to the manufacturer's instructions. They were analyzed within 1 h by flow cytometry using a FACSArray Apparatus (BD Biosciences) and CellQuest (BD Biosciences) and ModFit LT (Verify software, Topsham, MN) software.

##### Caspase-3 assay

The activity of caspase-3 was measured in whole cell lysates pre-treated or not with either 10^-7 ^M cytochalasin B, or 10 μM Y-27632 and then stimulated with 10^-7 ^M testosterone-BSA for the time periods indicated in the figure legends, using the Clontech ApoAlert^® ^Caspase Colorimetric Assay kit according to the manufacturers' instructions. Caspase-3 activity was determined by incubating lysates with a caspase-3 substrate (the peptide DEVD conjugated to the chromophor p-nitroaniline) for 2 h at 37°C. The absorbance of each sample was measured at 405 nm by using a 96-well colorimetric plate reader.

## Results

### Testosterone and testosterone-albumin-conjugates trigger similarly specific activation of mAR in DU145 cells

**Figure 1:**
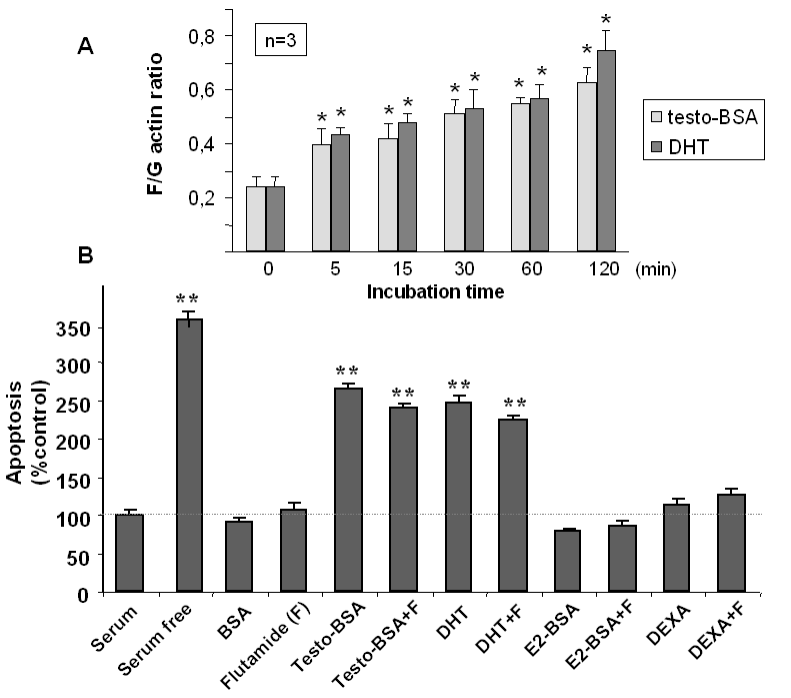
**Modulation of the dynamic equilibrium between G- and F- actin and induction of apoptosis in testosterone-BSA stimulated DU145 cells**. A) 24 h serum starved cells were stimulated with 10^-7 ^M testosterone-BSA or DHT for the indicated time points. F- and G- actin were measured by quantitative immunoblot analysis after Triton X-100 subcellular fractionation. Bars represents the F/G actin mean value ± SE of three independent duplicate experiments (*P < 0.05). B) *APOPercentage Apoptosis Assay *of steroid-stimulated DU145 cells in the presence or absence of androgen receptor antagonist, flutamide (F). Cells pre-treated or not with 10^-7 ^M flutamide (F), were incubated with testosterone-BSA, dihydrotestosterone (DHT), estradiol-BSA (E2-BSA), dexamethasone (DEXA) or control BSA dissolved in serum-supplemented medium at a final concentration of 10^-7 ^M for 24 hours. Cells serum starved for equal period of time served as a positive control for apoptosis. The mean OD measured at 550 nm ± SE of three independent experiments performed in triplicates was normalized to the mean OD ± SE of the untreated control (serum supplemented) cells. Results are presented in bars as percentage (%) of control cells taken as 100% (**P < 0.01).

As previously reported, DU145 cells are iAR-negative and express functional mAR [13,17,19]. Accordingly, we have evaluated early and late effects following mAR-activation by using both, free testosterone and testosterone albumin conjugates. Since actin reorganization is a main early cellular response triggered by testosterone-BSA in various tumor cell types [7,12,17], we first compared the rapid alterations of actin polymerization dynamics in testosterone-BSA- and DHT-treated DU145 cells. As shown in Figure 1A, DHT induced rapid-within 5 minutes- and sustained F/G-actin ratio increase, indicative for potent actin polymerization, exactly as shown for testosterone-BSA-treated DU145 cells (Fig 1A and ref. [17]). The F/G-actin ratio-increase kinetics (Fig 1A) were comparable for free and albumin-conjugated testosterone, indicating similar mAR-activation potential by DHT and testosterone-BSA. We have further evaluated the apoptosis of DU145 cells stimulated by DHT and testosterone-BSA. As shown in Figure 1B both androgen analogs induced similar apoptotic responses. As expected for an iAR-negative cell line, this effect was not abolished in the presence of the iAR antagonist flutamide in DU145 cells. Moreover, BSA itself was ineffective (Fig 1B). From these findings we can rule out nonspesific testosterone-BSA-induced mAR stimulation. Finally, we evaluated the steroid-hormone specificity of the mAR-induced apoptotic responses in DU145 cells. For this, we studied the induction of apoptosis by using BSA-conjugated estradiol and free dexamethasone. As shown in Fig. 1B, both steroid hormones could not generate any apoptotic response in DU145 cells, while testosterone-BSA showed clear activity. Taken together these results provide strong evidence that activation of mAR by testosterone or testosterone-conjugates is specific, triggering early and long-term downstream cellular responses in prostate cancer cells.

### Testosterone-BSA induces long term inhibition of PI-3K activity in DU145 cells

**Figure 2:**
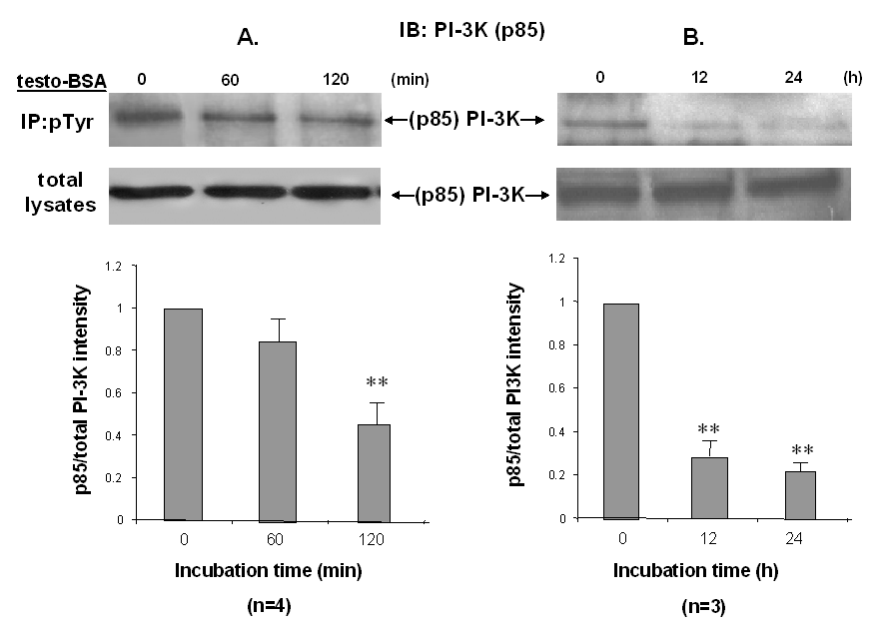
**Long-term dephosphorylation/inactivation of PI-3K in DU145 cells**. Serum supplemented DU145 cells were stimulated with 10^-7 ^M testosterone-BSA for the indicated time periods (A: 0, 60 or 120 min and B: 0, 12 or 24 h). Following cell lysis equal amounts of proteins were immunoprecipitated (IP) with an anti-phosphotyrosine (pTyr) antibody. The tyrosine-phosphorylated as well as equal amounts of total lysates were immunoblotted (IB) with a specific antibody against the p85 subunit of PI-3K. The immunoblots were analyzed by densitometry. The intensity of phosphorylated PI-3K bands was normalized to the intensity of the corresponding total PI-3K band. Blots show a representative experiment, while the relative fold decrease in (p85)PI-3K phosphorylation with that of untreated cells taken as 1 are shown in the chart. Each bar represents mean values ± SE of at least three independent experiments (**P < 0.01).

In contrast to iAR^+ ^LNCaP cells, testosterone-BSA-stimulated iAR^- ^DU145 cells fail to rapidly activate FAK/PI-3K signaling [8,17]. To evaluate the long term activity of PI-3K and its possible involvement in the apoptotic response, we treated DU145 cells with 10^-7 ^M testosterone-BSA for various time periods and evaluated the tyrosine phosphorylation activity of the p85 regulatory subunit in IP-Western assays (Fig 2). Total cell lysates subjected to Western blot analysis with p85 antibodies were used as controls. Testosterone-BSA significantly inhibited p85-activity already 2 h post stimulation (Fig 2A). Further exploration of the time-dependent effect of testosterone-BSA on PI3K activity showed almost maximal inhibition within 12 h, which sustained up to 24 h (Fig 2B). These results indicate that stimulation of DU145 cells by testosterone-BSA results in long term dramatic suppression of PI-3K activity, a finding compatible with the reported mAR-induced apoptosis of this cell model.

### Testosterone-BSA inhibits long term Akt activity and induces Bad de-phosphorylation in DU145 cells

**Figure 3:**
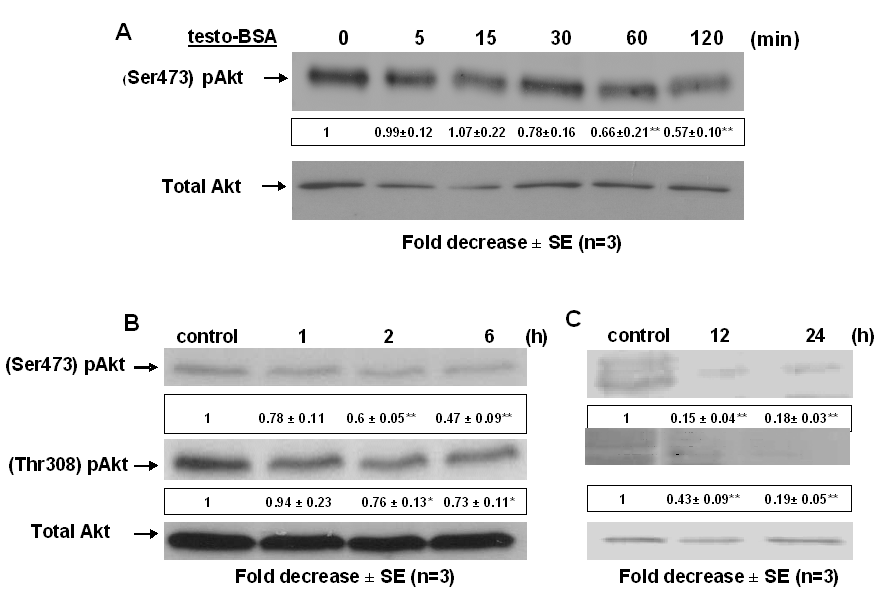
**Long-term dephosphorylation/inactivation of Akt kinase in DU145 cells**. DU145 cells were exposed to 10^-7 ^M testosterone-BSA for the indicated time periods: (A: 0, 5, 15, 30, 60 and 120 min), (B: 0, 1, 2, 6 h) and (C: 0, 12 and 24 h). The ratio of the cellular content of the phosphorylated (at Ser473 or Thr308 residues) versus the total isoform of Akt kinase was measured in cell lysates by Western blotting using specific antibodies for each form and was normalized to the corresponding ratio of control. Blots show a representative experiment, while the numbers below each lane correspond to the mean values ± SE from three independent experiments (*P < 0.05, **P < 0.01) indicating the fold-decrease in the phosphorylation level for the indicated time point normalized to the corresponding short- and long-term untreated controls, respectively.

Akt/PKB is a serine/threonine kinase frequently activated in prostate cancer by various mechanisms including phosphorylation by PI-3K or loss of expression/activity of the PTEN tumor suppressor [23-25], reviewed in [26]. Activated Akt is a pro-survival factor controlling phosphorylation and activity of various pro-apoptotic gene products including caspase 9 [27] and Bad [28]. Since PI-3K activates Akt/PKB by phosphorylation at both the catalytic subunit (Thr 308) and its C-terminus (Ser 473) [29-31] and mAR-induction suppresses long term PI-3K activity in DU145 cells (Fig 2), we sought to identify the activity status of Akt in cells treated with testosterone-BSA for different time periods. Figure 3A shows that Akt(Ser473) phosphorylation, although it remained unaffected in the early phase, it decreased by almost 50% after a 2 h testosterone-BSA treatment. Long-term incubation experiments fully supported this finding. Indeed, Akt phosphorylation at Thr 308 and Ser 473 is significantly decreased starting from 2 h (Fig 3B), and is almost completely suppressed after 24 h testosterone-BSA treatment (Fig 3C) following very similar kinetics to the (p85)PI-3K inhibition. Taken together these results indicate that testosterone-BSA down-regulates the activity of the PI-3K/Akt pro-survival pathway in DU145 cells.

**Figure 4:**
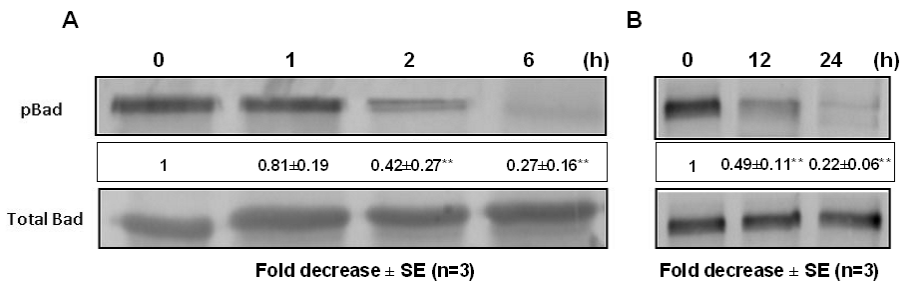
**Testosterone-BSA induces activation of the pro-apoptotic Bad protein in DU145 cells**. DU145 cells were exposed for different time periods (A: 0, 1, 2, 6) and (B: 12 and 24 h) to 10^-7 ^M testosterone-BSA (testo-BSA). The ratio of the cellular content of the phosphorylated versus the total form of Bad was determined in cell lysates by Western blotting using specific antibodies. Blots show a representative experiment, while results are presented as the mean values ± SE from three independent experiments normalized versus the corresponding short- and long-term untreated controls respectively (**P < 0.01).

In agreement with the inhibition of Akt activity shown in Fig 3 and based on the well-documented role of this kinase in inactivating the pro-apoptotic function of Bad via phosphorylation [28,32], we further analyzed the activity of Bad. Figure 4 clearly shows that testosterone-BSA treatment results in dephosphorylation/activation of Bad following the same pattern as Akt [statistically significant reduction of phosphorylation begins at 2 h (Fig 4A), reaching minimum levels of phosphorylated Bad at 24 h (Fig. 4B)]. From this finding we conclude that Bad activation contributes to the testosterone-BSA-induced apoptotic events in DU145 cells.

### Testosterone-BSA suppresses NF-κB activity in DU145 cells

**Figure 5:**
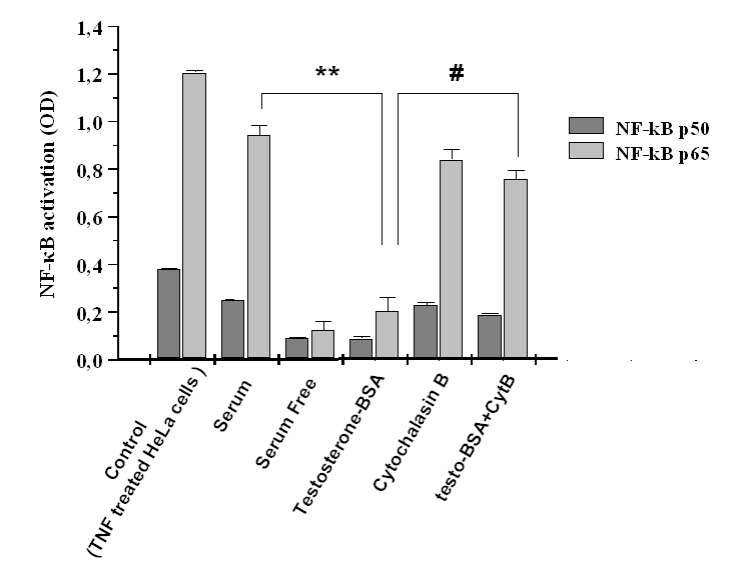
**Testosterone-BSA suppresses the activity of the transcription factor NF-κB, which is reversed by the actin inhibitor cytochalasin B**. DU145 cells grown in serum supplemented medium were exposed for 60 min to 10^-7 ^M testosterone-BSA, or to 10^-7 ^M cytochalasin B alone or in combination with testosterone-BSA. TNF-treated HeLa cells or serum supplemented DU145 cells were used as positive controls, 24 h-serum starved DU145 cells as negative control respectively. At the end of the incubation NF-κB activity was measured as described in Materials and Methods. Results are normalized to NF-κB activity in cells cultured in serum-free medium in the absence of steroids and are presented as the mean values ± SE of four independent experiments performed in triplicates (**P < 0.01 for testosterone-BSA treatment versus serum conditions, and ^#^P < 0.01 for testosterone-BSA treatment alone versus testosterone-BSA treatment plus cytochalasin B).

NF-κB is normally localized in the cytoplasm as an inactive complex through physical association with its inhibitory molecule IκB (reviewed in [33]). Work from several laboratories has determined a sequence of biochemical events resulting in the ubiquitin-dependent degradation of IκB proteins [34-36], translocation of NF-κB into the nucleus and activation of anti-apoptotic and pro-inflammatory genes [37,38]. To address the functional implication of NF-κB in mAR-signaling, we treated DU145 cells with 10^-7 ^M testosterone-BSA for 1 h and measured NF-κB activity using a kit based on NF-κB's nuclear translocation as described in Materials and Methods. As becomes evident in Figure 5, testosterone-BSA treatment inhibits NF-κB activity in DU145 cells in agreement with the capacity of testosterone-BSA to induce apoptosis by down-regulating several pro-survival pathways in prostate cancer cells. Interestingly, inhibition of NF-κB activity was abolished in the presence of the actin-disrupting drug cytochalasin B confirming the recently postulated regulatory role of actin reorganization in mAR-induced apoptosis in prostate cancer cells [17].

### Testosterone-BSA induces FasL expression in DU145 cells

**Figure 6:**
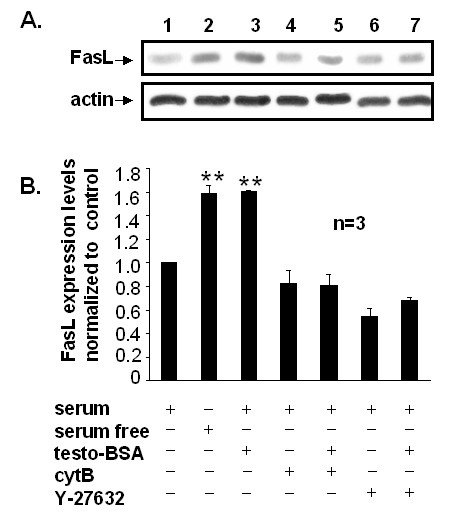
**Testosterone-BSA induces FasL expression, which is inhibited by the actin cytoskeletal drug cytochalasin B and the specific ROCK inhibitor Y-27632**. (A) Cells were pre-treated (+) or not (-) with 10^-7 ^M cytochalasin B (cyt B) or Y-27632 (10 μM) for 1 h, and then exposed (+) or not (-) to 10^-7 ^M testosterone-BSA in serum supplemented medium. 24 h serum starved cells served as a positive control for apoptosis. (A) Representative experiment showing FasL expression levels determined by immunoblotting equal amounts of cell lysates with a specific anti-FasL antibody (upper panel). The membrane was then stripped and re-probed with a specific anti-actin antibody to ensure equal protein loading (lower panel). Immunoblots were analyzed by densitometry. The relative fold increase in FasL expression with that of untreated cells taken as 1 is shown in the chart. Each bar represents mean values ± SE of three independent experiments (**P < 0.01).

**Figure 7:**
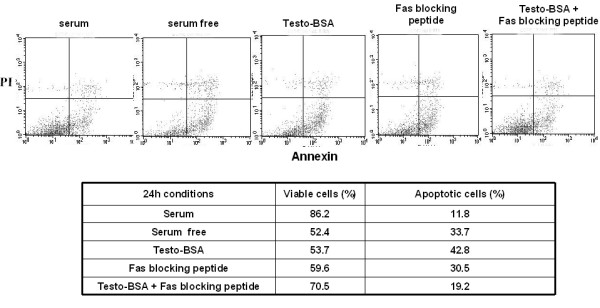
**Testosterone-BSA-induced apoptosis is inhibited in the presence of a specific Fas blocking peptide**. FACS analysis of DU145 cells treated with 10^-7 ^M testosterone-BSA (serum conditions), 10 μM of Fas blocking peptide (in serum free conditions) and both of these in parallel (serum conditions). Cells cultured in serum or 24 h serum starved cells were used as a negative and positive control for apoptosis respectively. Cells were co-stained with Annexin V-FITC for apoptotic cells and the vitality marker propidium iodide (PI). Representative percentages of viable and apoptotic cells under each condition are presented in the table.

Having established a clear role for testosterone-BSA in suppressing the activity of PI-3K/Akt and NF-κB pro-survival pathways in DU145 cells, we sought to characterize the mechanism of apoptotic induction by testosterone-BSA. Since the CD95/FasL death pathway was recently shown to be involved in testosterone-BSA-induced apoptosis in LNCaP cells [13], we further investigated whether Fas receptor activation is evident in DU145 cells. Figure 6A, B shows Western blot analysis and corresponding quantitative determinations of pro-apoptotic FasL protein in DU145 cells treated with testosterone-BSA for 6 h. Our results show a significant increase in FasL expression. Interestingly, this induction was abolished in cells pre-treated with the actin cytoskeleton disruptor cytochalasin B (Fig 6A, compare lanes 3 and 5), or with the ROCK inhibitor Y-27632 (Fig 6A, compare lanes 3 and 7), indicating a regulatory role for actin and Rho/ROCK signaling in FasL expression. To confirm the participation of the Fas/FasL pro-apoptotic effectors in testosterone-BSA-induced apoptosis, we measured its apoptotic effect in the presence of a specific Fas blocking peptide. Although the Fas blocking peptide was unexpectedly shown to induce apoptosis on its own (eventually through interaction with the Fas receptor), it completely reversed the apoptotic effect of testosterone-BSA (Fig 7). These results suggested that testosterone-BSA-mediated up-regulation of FasL and the subsequent activation of Fas receptor by its ligand are the predominant mechanisms regulating cell death in DU145 cells. From these findings and the activation of Bad evident in Fig 4, we concluded that FasL and Bad mediate the testosterone-BSA-induced apoptotic events in DU145 cells.

### mAR-stimulation by testosterone-BSA triggers caspase-3 activation in DU145 cells

**Figure 8:**
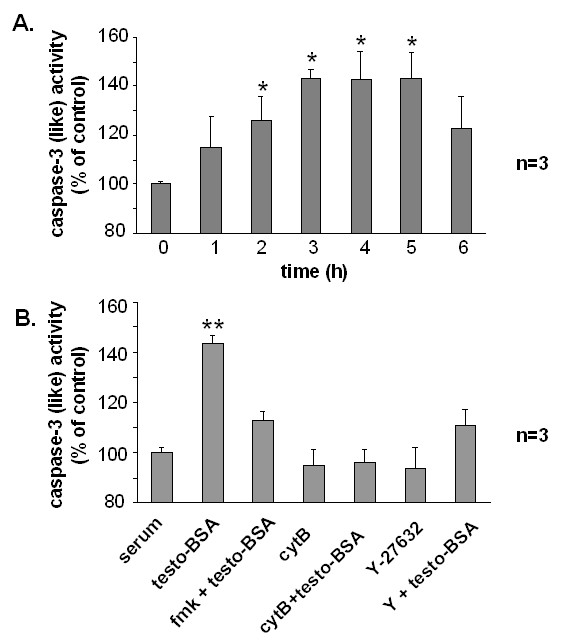
**Testosterone-BSA induces Caspase-3 activation**. A) Caspase-3 activity was measured at 405 nm in lysates derived from cells exposed to 10^-7 ^M testosterone-BSA for the indicated time points and then incubated with the caspase-3 substrate DEVD conjugated to the chromophor pNA as described in Methods and Materials. B) Cells were pre-treated or not with 10^-7 ^M of cytochalasin B or Y-27632 (10 μM) for 1 h and then exposed or not to 10^-7 ^M testosterone-BSA for 3 h. The relative caspase-3 activity is expressed as percentage with that of serum cultured cells taken as 100%. Data presented in bars are mean values ± SE of three independent experiments (*P < 0.05).

To assess the participation of caspases as executors in mAR-dependent cell death, we measured caspase-3 activity in testosterone-BSA-treated DU145 cells. As presented in Fig 8A, activation of caspase-3 became evident within 2 h upon testosterone-BSA treatment, reaching maximal activity after 3–5 hours. This effect was blocked by the caspase-3 inhibitor DEVD-fmk, or in cells pre-treated with cytochalasin B or with the ROCK inhibitor Y-27632 (Fig 8B). These results indicate that in mAR-stimulated DU145 cells, caspase-3 activation is mediated by actin redistribution through the Rho/ROCK pathway.

## Discussion

Previous studies in prostate cancer cell lines have established a clear role for membrane androgen receptors in the induction of apoptotic responses via actin cytoskeleton reorganization [17]. Furthermore, it has been proposed that specific mAR-activating ligands, namely testosterone-serum albumin conjugates, may be developed as novel drug candidates for the treatment of mAR+ prostate tumors [17,18].

Using pharmacological inhibitors, dominant negative alleles and various functional assays, we were able to identify in previous studies a series of key denominators of mAR function in prostate cancer cell lines [8,17]. Specifically, we have identified a FAK/PI-3K/Rac/Cdc42 pathway triggered by testosterone-BSA in LNCaP cells, resulting in actin cytoskeleton reorganization [8]. In DU145 cells FAK and PI-3K were shown to be constitutively active, and testosterone-BSA triggers actin rearrangements via a Rho/ROCK/LIMK2/ADF-destrin signaling pathway [17]. Interestingly, the same Rho/ROCK pathway operates in LNCaP cells, downstream of FAK/PI-3K/Rac1 [17]. Finally, actin cytoskeleton disrupting agents and ROCK inhibitors were shown to block mAR-dependent apoptosis in both cell lines, indicating that Rho/ROCK/actin signaling is a key regulator of apoptotic responses [17]. However the identification of the specific downstream players implicated in apoptosis remained unknown.

In the present work we characterized the specificity of mAR by using a series of BSA-conjugated and free steroid hormones, providing clear evidence for testosterone specificity. We further explored the mechanism of cell death triggered by mAR-stimulation by analyzing the expression and activity of several gene products and pathways involved in the regulation of survival and apoptosis of DU145 prostate cancer cells. Using testosterone-BSA as a specific mAR ligand, we show here that mAR activation results in almost complete down-regulation of the activity of PI-3K, Akt and NF-κB in DU145 cells. Concurrently, testosterone-BSA induces FasL expression, activates Bad and up-regulates the activity of caspase-3, indicating that mAR-stimulation affects prominent pro-apoptotic regulators [39,40]. Importantly, these effects are blocked by actin cytoskeleton disrupting agents or the ROCK inhibitor Y-27632, providing additional evidence that actin reorganization, shown to be a prominent event in mAR-stimulated prostate cancer cells [Fig 1A and ref [7,8,41]], and the newly identified Rho/ROCK signaling [17] control testosterone-BSA-induced apoptosis in DU145 cells. These data further corroborate the hypothesis postulated by several research groups that actin dynamics reorganization is a key regulator of apoptotic responses (for reviews see [42-44]). Taken together our results offer further mechanistic insights into the control of survival and apoptosis downstream of mAR, pointing to specific pro-apoptotic molecular effectors acting most probably downstream of Rho/Rock/actin.

Although we cannot rule out that the observed changes in the expression and activity of all analyzed proteins are the consequence rather than the cause of mAR-dependent apoptosis, the data clearly underscore the key role of mAR-activating ligands in the selective elimination of DU145 cells. Moreover, these receptors are specific for testosterone and testosterone-albumin conjugates, since other steroid hormones-conjugated or not-failed to exhibit any pro-apoptotic activity. Notably, these cells typically represent an aggressive pre-clinical hormone-refractory cell line model used to assess the anti-tumor ability of chemotherapeutic drugs, as they (I) are devoid of functional intracellular androgen receptors (iARs) and (II) fail to respond to androgen treatment [19]. Based on these results, mAR may be a novel target that can be used for the selective elimination of mAR^+ ^prostate cancer cells independently of the functional status of the intracellular androgen receptor. Interestingly, mAR is selectively over-expressed in biopsy samples from aggressive, high-Gleason prostate tumors in comparison to samples from benign prostate hyperplasia patients or healthy subjects [45,46].

Future experiments will focus on the identification of additional signaling targets downstream of mAR and the characterization of functional synergies of mAR-dependent signals with other pathways activated in prostate cancer. Characterization of the functional interplay between membrane and intracellular androgen receptors may contribute to the understanding of the apparent discrepancy in the actions of androgens inducing both proliferation and death within a given cell. Our present findings elucidating at least parts of the mAR-induced molecular pro-apoptotic machinery in DU145 cells provide novel insights in membrane GPCR mediated non-genomic androgen actions.

## Competing interests

The authors declare that they have no competing interests.

## Authors' contributions

NP and IC carried out the analysis of all signaling molecules, apoptotic responses and actin cytoskeleton dynamics. VA and GK carried out the kinetics of pro-survival gene products and FACS analysis. MF participated in the design of the study and performed the statistical analysis. KA participated in the design of the study and drafted the manuscript. FL and AG participated in the coordination of the study and evaluation of the results. CS conceived of the study and participated in the design and coordination.
